# Prevalence of psoriasis in indigenous communities around the world: a scoping review^⋆^^؆^

**DOI:** 10.1016/j.abd.2026.501421

**Published:** 2026-08-12

**Authors:** Yebin Yang, Sangho Lee, Francis Yi Xing Lai, Ricardo Romiti

**Affiliations:** a Department of Dermatology, Monash Health, Victoria, Australia; b Skin Health Institute, Victoria, Australia; c Department of Dermatology, Hospital das Clínicas, Universidade de São Paulo, SP, Brazil

**Keywords:** Health of indigenous peoples, Incidence, Native people, Prevalence, Psoriasis, Skin pigmentation

## Abstract

Psoriasis is a chronic inflammatory condition with systemic comorbidities and significant impacts on quality of life. Its prevalence in indigenous populations is poorly understood, limiting targeted health interventions. This scoping review aims to review the prevalence of psoriasis in indigenous communities globally, identify genetic, environmental, and socioeconomic influences, and explore barriers to accurate diagnosis and care. A search of PubMed, Ovid MEDLINE, Cochrane Library, and Scopus identified 30 studies, of which 15 met inclusion criteria, encompassing 13 Indigenous populations across six continents. Almost all indigenous groups exhibited markedly lower psoriasis prevalence compared to global estimates, with zero prevalence reported in the Taiwan Ami, Tanzanian Maasai, and Aboriginal groups in Brazil and Peru, and anecdotally rare in Aboriginal people of Australia, Native Alaskans, First Nation Canadians, and Native Americans. The majority of indigenous populations demonstrated a lower prevalence of psoriasis than their respective national population. Factors including protective genetic and environmental triggers, degrees of ultraviolet exposure, environmental influences, and cultural and traditional lifestyles are postulated explanations. However, limited access to specialists, diagnostic challenges in skin of colour, and cultural differences impede accurate estimation of prevalence. Standardised research methodologies and culturally sensitive healthcare strategies are crucial to address disparities and improve recognition in these communities.

## Introduction

Psoriasis is a chronic, multi-system inflammatory skin condition associated with psoriatic arthritis, metabolic comorbidities and major cardiovascular events, resulting in a substantial reduction in quality of life and significant impact on mental health.^1^ In 2016, the World Health Organization recognised psoriasis as a serious non-communicable disease, necessitating efforts to accurately define its global prevalence to guide targeted health policies.^1^

Global prevalence figures vary largely, from 0.4% to 8.5%.^2^ Considerable variation exists across age, sex, ethnicity, and socioeconomic groups, even within the same geographical region.^3^ Indigenous peoples, defined as groups who self-identify as distinct communities with ancestral ties to historical populations of a region, remain underrepresented in medical research.^4^, ^5^ Limited public health surveillance further obscures accurate estimates of disease burden, leaving the true extent of health disparities largely unknown. However, several individual studies suggest that indigenous communities have a lower prevalence of psoriasis compared with non-indigenous populations.

This scoping review aims to synthesise the existing evidence on psoriasis prevalence in Indigenous populations globally and compare against the national psoriasis prevalence of the corresponding countries. The authors also explore the genetic, environmental, geographical, and socioeconomic determinants of disease, and highlight public health barriers that hinder accurate assessment of health burden on indigenous communities.

## Methods

The International Prospective Register of Systematic Reviews (PROSPERO) review protocol was created (registration number CRD420251075399) outlining the eligibility criteria, information sources, search strategy and data analysis.

**Fig. 1:**
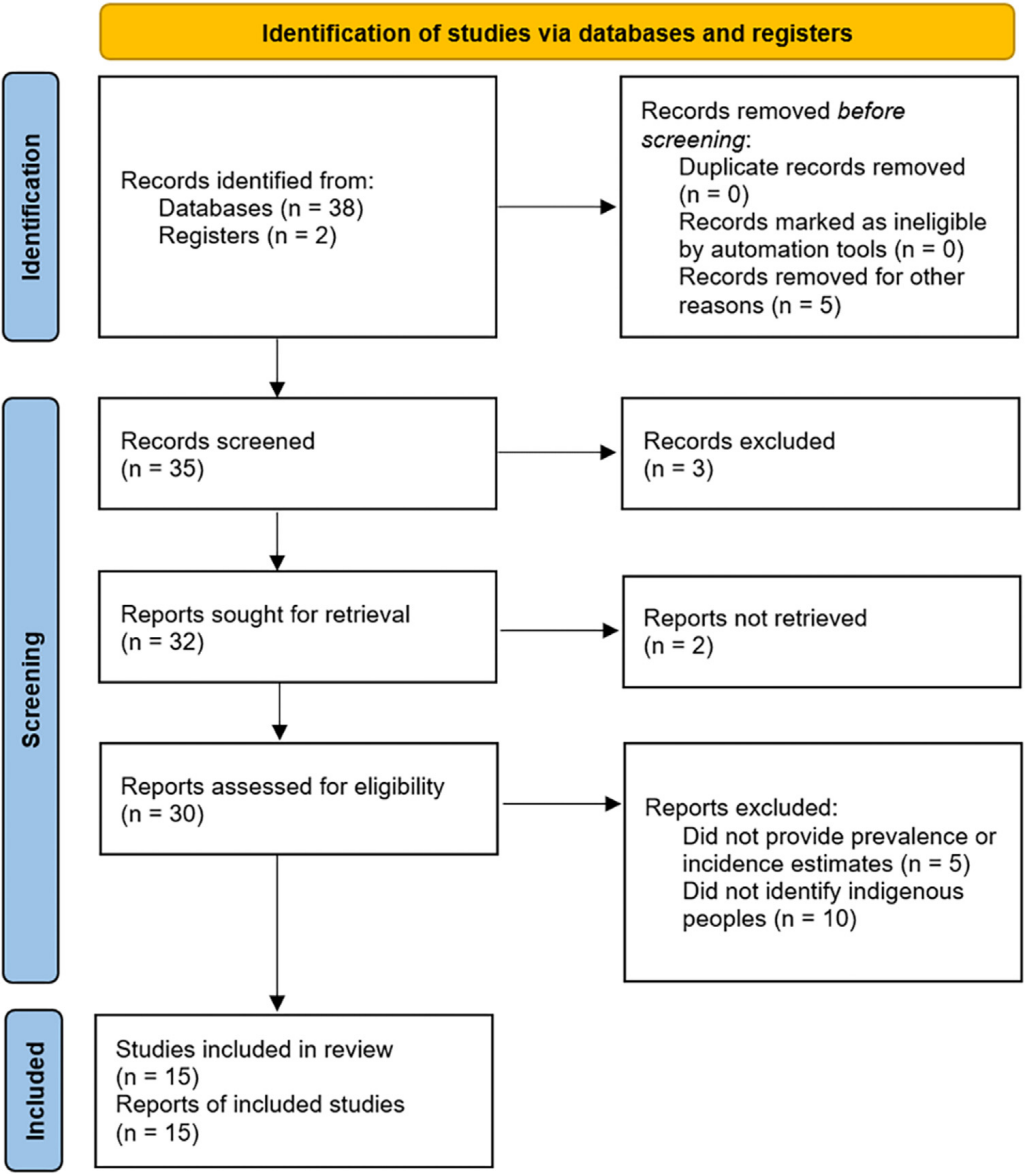
Preferred Reporting Items for Systematic reviews and Meta-Analyses (PRISMA) flowchart for identification, screening and inclusion of studies.^6^.

A scoping search was conducted in PubMed, Scopus, Cochrane Library, and MEDLINE (via OVID) using a combination of terms “psoriasis”, “indigenous”, “native”, “Aboriginal”, “population groups”, “epidemiology” and “prevalence” with no date restrictions. One of the electronic search strategies used is as follows: (“Psoriasis”[Mesh] OR psoriasis) AND (“Indigenous Peoples”[Mesh] OR indigenous OR aboriginal OR native OR First Nations) AND (“Epidemiology”[Subheading] OR epidemiology OR “Prevalence”[Mesh] OR prevalence). Studies were eligible for inclusion if they reported the prevalence of psoriasis in Indigenous communities of any age. Articles were excluded if they did not provide prevalence estimates, unless they represented the only available data for a given Indigenous group. A Preferred Reporting Items for Systematic reviews and Meta-Analyses (PRISMA) flowchart summarises the number of studies identified, screened, and included for this scoping review in Fig. 1.

Titles and abstracts were screened independently by two investigators (YY and SL). Discrepancies were resolved through discussion and consensus. The process identified 30 publications addressing psoriasis prevalence in Indigenous populations worldwide. References of included studies were also reviewed to capture additional eligible articles. Following full-text review, 15 studies were excluded for not reporting Indigenous-specific data or for lacking prevalence figures, leaving 15 studies for inclusion. A study that identified indigenous-specific data but reported incidence of psoriasis instead of prevalence was determined to provide valuable insight, aligning with the objectives of the review and was included for extraction.

Data extraction included predefined quantitative and qualitative variables relating to psoriasis prevalence. Quantitative variables comprised the number of individuals diagnosed with psoriasis (numerator), total population size (denominator), and calculated or reported prevalence estimates. Where available, subgroup-specific data (e.g., by region) were also extracted. Qualitative variables included study setting, population characteristics, and diagnostic criteria used to define psoriasis.

To contextualise findings, an additional PubMed search was performed using the terms psoriasis, prevalence, and the names of countries of origin of the included Indigenous groups to identify corresponding national prevalence estimates for comparison.

**Fig. 2:**
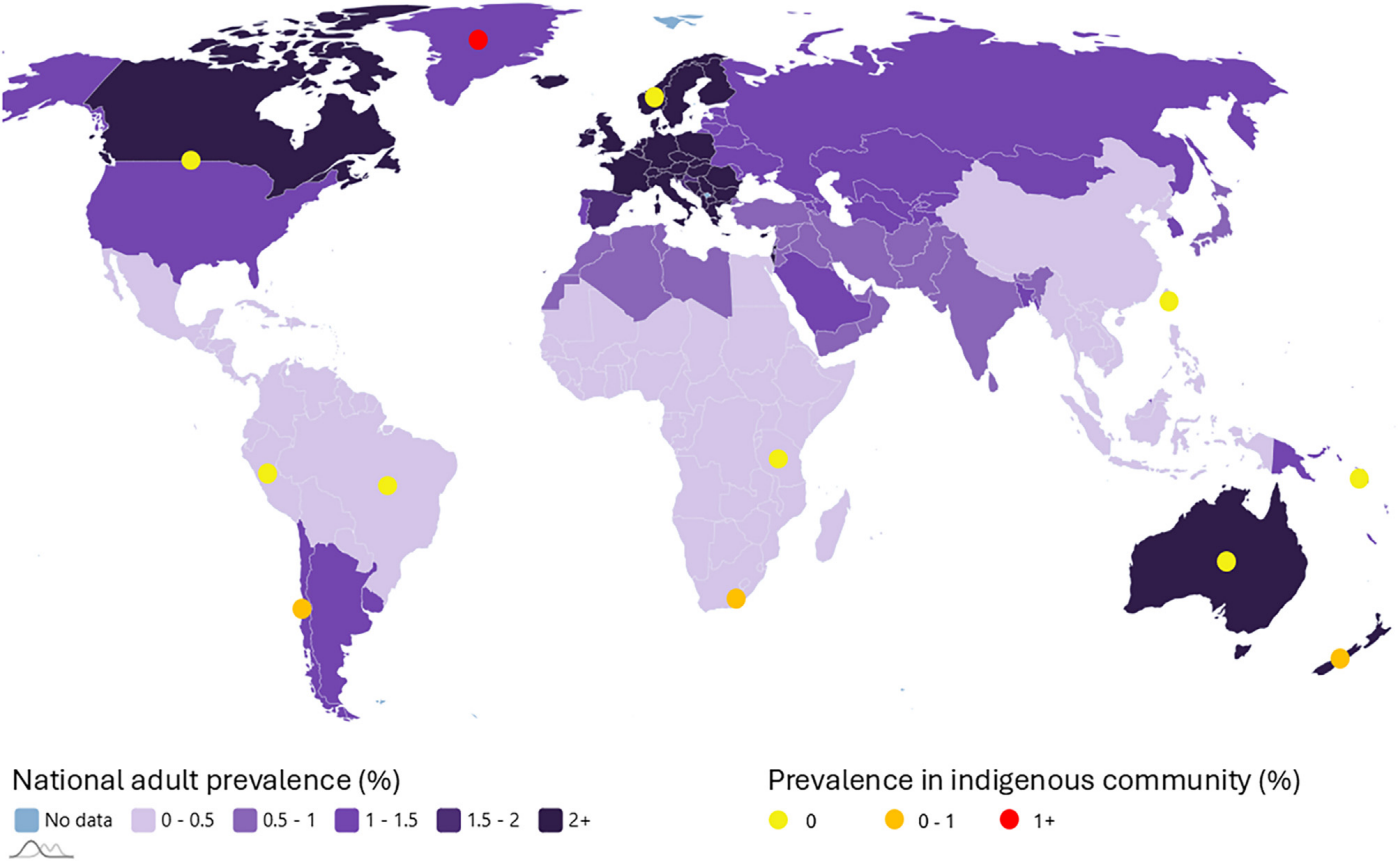
2026 Global Psoriasis Atlas prevalence heat map overlayed by psoriasis prevalence of indigenous communities in their respective geographical locations.^23^, ^24^.

Results were extracted and summarised in table format available in Table 1. Fig. 2, which demonstrates psoriasis prevalence of indigenous communities compared to their corresponding national adult prevalence, was produced to further visualise the geographical distribution and the limited number of indigenous communities with known psoriasis prevalence estimates. The ‘Psoriasis Prevalence Heat Map’ was extracted from the Global Psoriasis Atlas via exporting a PNG of the map from the website, which was imported into Microsoft PowerPoint (Version 2603 Build 19822.20150 Click-to-Run) for the addition of legend titles, overlaying coloured circles representing disease prevalence in indigenous communities, and a corresponding legend key.

**Table 1:** Prevalence of psoriasis in indigenous groups compared with corresponding national populations.^2^, ^3^, ^7^, ^8^, ^9^, ^10^, ^11^, ^12^, ^13^, ^14^, ^15^, ^16^, ^17^, ^18^, ^19^, ^20^, ^21^, ^22^, ^23^, ^24^, ^25^, ^26^, ^27^, ^28^, ^29^, ^30^, ^31^, ^32^.

Continent	Country and indigenous community	Psoriasis prevalence (%)	Number of cases / total number of people in study (n)	National adult psoriasis prevalence, if reported (%)	Reference
Asia	Taiwan ‒ Ami people	0	Anecdotal	0.18 ‒ 0.86	Chandran,^7^ Bos,^19^ Heyes;^32^ Iskandar,^20^ Global Psoriasis Atlas^23^, ^24^
Oceania	Australia ‒ Aboriginal people	“Extremely rare”	Anecdotal	2.38 – 3.78	Green,^8^ Heyes;^32^ Parisi,^3^ Foley,^21^ Global Psoriasis Atlas^23^, ^24^
	American Samoa ‒ Samoan People	0	0/12,569	0.34 – 1.13	Valenzuela;^9^ Jacobson,^31^ Global Psoriasis Atlas^23^, ^24^
	New Zealand ‒ Maori people in Auckland Health District Board	0.14	52/∼36630	2.50	Lee,^18^ Health Board;^22^ Global Psoriasis Atlas^23^, ^24^
Europe	Greenland ‒ Inuit people in Tasiiliq	5.7	16/283	1.1 ‒ 1.34	Haulrig;^10^ Global Psoriasis Atlas,^23^, ^24^ Botvid^29^
	Norway ‒ Sami people	1.4	35/2508	3.12 ‒ 4.6	Chandran;^7^ Global Psoriasis Atlas,^23^, ^24^ Solberg^25^
North America	Canada and the United States ‒ Native Alaskan, First Nation Canadian and the Native Americans	“Rare”	Anecdotal	United States: 1.12 ‒ 3.0	Raychaudhuri;^11^ Armstrong,^2^ Parisi,^3^ Global Psoriasis Atlas^23^, ^24^
				Canada: 1.34 – 2.65	
South America	Brazil ‒ indigenous people	All indigenous groups in Brazil ‒ 0.0012	All indigenous groups in Brazil ‒ 953/775,995	0.30 ‒ 1.31	Romiti;^12^ Global Psoriasis Atlas,^23^, ^24^ Romiti,^26^ Andrade^30^
		Auaris region ‒ 0	Indigenous people in the Auaris region ‒ 0/555		
	Peru‒ Aboriginal people in the Andean regions	0	0/25,915	0.40 ‒ 2.5	Chandran,^7^ Toloza,^13^ Toloza;^14^ Global Psoriasis Atlas,^23^, ^24^ Rodriguez-Zuniga^27^
	Chile ‒ Mapuche people	0.26	1/380	1.43	Valenzuela;^9^ Global Psoriasis Atlas^23^, ^24^
Africa	Ethiopia ‒ Tigray People	‒	954 cases; incidence 183 per year	0.22	Morrone;^15^ Global Psoriasis Atlas^23^, ^24^
	Moshi, Tanzania ‒ Maasai people	0	0/51	0.15	Morrone,^9^ Khan;^16^ Global Psoriasis Atlas^23^, ^24^
	Eastern Cape (Mtyholo Dlova and Mdolomba), South Africa	0.7	5/698	0.39 ‒ 2.9	Wright;^17^ Hartshorne,^28^ Global Psoriasis Atlas^23^, ^24^

References for indigenous psoriasis prevalence and national adult psoriasis prevalence is divided by a semicolon (;).

* Pool of participants are not completely indigenous people, further data unable to be found.

## Results

There were 15 sources of evidence used to extract descriptions and numerical data on psoriasis prevalence for 13 Indigenous population groups across six continents (Table 1). For comparison, national psoriasis prevalence estimates of the corresponding countries were also included. Indigenous population sizes ranged from 300 to 36,650 individuals. Only anecdotal descriptions were available without an associated population denominator for the Taiwan Ami, Aboriginal Australians, and North American Indigenous peoples.^7^, ^8^, ^11^, ^19^^,^^32^ The Maori population size within the Auckland District Health Board was estimated using a 2014 report from the Board.^22^

In almost all cases, indigenous groups demonstrated a lower prevalence of psoriasis compared to their respective adult national populations. Complete absence of disease was reported among the Taiwan Ami people, American Samoan people, indigenous people in the Auaris region in Brazil, Andean Aboriginal people of Peru, and the Maasai people of Tanzania.^9^, ^13^, ^14^, ^15^, ^16^, ^31^ Anecdotal accounts also described psoriasis as extremely rare among North American Indigenous groups (Native Alaskan, First Nations Canadians, and Native Americans) and Aboriginal Australians.^4^, ^5^, ^8^, ^16^^,^^29^ Whilst prevalence was not yet determined, psoriasis incidence amongst the Tigray people of Ethiopia was reported to be 183 cases per year.^15^ The Inuit people in Tasiiliq, Greenland, were the only indigenous group to demonstrate a higher psoriasis prevalence (5.7%) compared to the adult national prevalence (1.1%‒1.34%).^10^, ^23^, ^29^

The highest disease prevalence was observed among Arctic Indigenous groups, with 5.7% in Inuit communities in Tasiilaq, Greenland, and 1.4% in the Sami people of Norway.^7^, ^10^ This was followed by 0.7% among Eastern Cape populations in South Africa, 0.26% within the Mapuche people in Chile, and 0.14% in the Maori people in New Zealand.^9^, ^20^, ^21^, ^22^, ^29^

By continent, psoriasis was most common among Arctic indigenous groups in Europe (Inuit people in Greenland and Sami people in Norway) with a weighted average of 1.15%.^7^, ^10^ Asia showed the lowest prevalence overall as a continent, although findings were limited with the inclusion of a single indigenous group. These trends are comparable to trends found in non-indigenous populations, where the highest rates were seen across Europe, Oceania, and North America (Norway 3.12%‒4.6%, Australia 2.38%‒3.78%, New Zealand 2.5%, Canada 1.34%‒2.65%, United States 1.12%‒3.0%).^3^, ^7^, ^21^, ^23^^,^^25^

The prevalence of indigenous groups compared to their respective non-indigenous national prevalence is shown in Fig. 2.

## Discussion

Most indigenous populations were found to have lower prevalence rates of psoriasis, compared to their non-indigenous counterpart communities, with prevalence ranges of 0.15%–3.78%, with some reporting complete absence of the disease.^23^ Various biological, environmental, and sociocultural factors have likely influenced the course of the disease for these populations.

### Genetic susceptibility and epigenetics

The pathogenesis of psoriasis is closely linked to dysregulation of innate and adaptive immune pathways.^1^ Epigenetic mechanisms may further explain population-level differences in psoriasis prevalence, as environmentally driven changes in gene expression can modulate immune responses without altering genetic background.^33^, ^34^, ^35^ One of the most significant genes of interest is the HLA-Cw6 allele, which has a strong association with psoriasis.^4^, ^7^, ^9^, ^30^, ^31^, ^32^, ^33^, ^34^, ^35^, ^36^, ^37^, ^38^, ^39^ HLA-Cw6 is more common in Caucasian patients than in Asian patients, with the presence of HLA-Cw6 conferring a relative risk of 13 for developing psoriasis in the Caucasian population compared to 25 in the Japanese populations.^37^ Importantly, genetic predisposition appears to be a stronger determinant than geographic location of indigenous groups. Despite their large geographical distance, North American Indians, Aboriginal Australians and the Taiwan Ami people- three indigenous groups that descended from the same ancestor- all have a negligible prevalence compared to non-indigenous populations living in the same areas.^3^, ^32^

Epigenetics provides a mechanistic link between genetic susceptibility and environmental exposures in psoriasis. Through processes such as DNA methylation, histone modification, and microRNA regulation, external stimuli can induce persistent changes in gene expression that influence key inflammatory pathways, including the IL-23/Th17 axis and keratinocyte proliferation.^33^, ^34^ Therefore, in genetically predisposed populations, environmental triggers may promote pro-inflammatory modifications that unmask disease expression- conversely, this means in populations without risk alleles, environmental exposures alone may be insufficient to induce the changes required to trigger disease.^33^, ^34^, ^35^ This may help explain the persistently low prevalence of psoriasis observed in certain indigenous groups despite increasing exposure to similar environmental risk factors as non-indigenous populations currently.

### Colonisation and exposure to infection

Studies have demonstrated a positive correlation between psoriasis incidence and heightened innate immune activity, which is hypothesised to have evolved from ancestral exposure to infectious diseases.^9^, ^14^, ^32^ The majority of indigenous groups with negligible psoriasis prevalence are thought to have experienced delayed exposure to streptococcal and other systemic infections compared to European populations. Evidence suggests that psoriasis was virtually absent in Australia until European settlement in the last 200-years, and similarly in Chile until the Spanish conquests in the 16^th^ and 17^th^ centuries, both of which introduced novel pathogens to these indigenous communities.^9^, ^14^, ^32^ Similar trends are reported among North American Indians and the Taiwan Ami, whose communities were also impacted by waves of colonisation in the 14^th^ to 16^th^ centuries.^3^, ^11^

A 1989 study investigating Human Leukocyte Antigen (HLA) and disease associations found that the major histocompatibility complex at HLA-A and HLA-B were nearly identical among indigenous Australians, consistent with an evolutionary history of reduced pathogen exposure and corresponding lower rates of psoriasis.^32^, ^36^ Such reduced variability likely restricts antigen presentation diversity, influencing how the immune system responds to environmental triggers implicated in psoriasis.^32^

### Environmental and lifestyle influences

Exposure to sunlight and UV radiation plays an important part in both disease modulation and therapeutic approaches to psoriasis. A systematic review of 85 papers demonstrated that psoriasis prevalence correlated with increasing distance from the equator, supporting the correlation between UV radiation exposure and clinical improvement.^21^ This pattern is reflected in our review, where most indigenous communities living closer to the equator, such as the Taiwan Ami, Indigenous Australians, American Samoans, and sub-Saharan African groups, reported no cases of psoriasis.^7^, ^8^, ^9^, ^14^, ^31^, ^32^^,^^40^ In contrast, Arctic Indigenous populations demonstrated higher prevalence rates, with 5.7% among Inuit people in Greenland and 1.4% among the Sami people in Norway.^7^, ^10^ Similarly, a study of the rural town of Arctic Kasach’ye demonstrated an outlier prevalence of 11.8%.^31^

Geographical factors such as altitude, seasonal sunlight availability, and surface reflectivity of snow and water contribute towards shaping the distribution of psoriasis. The widespread and persistent presence of snow cover in circumpolar regions is thought to enhance UV reflectivity and confer a protective effect against psoriasis.^31^, ^39^ It is also proposed that long-term biological adaptation to the high altitude and extreme cold may contribute to the lack of psoriasis in Peruvian Aboriginals in the Andean Mountains.^13^ A survey of 555 indigenous people in Brazil, which found no cases of psoriasis, also attributed its findings to the region’s humid and warm climate, high UV exposure, and minimal exposure to external and urban environmental triggers.^12^

Lifestyle factors such as diet, smoking and alcohol consumption, obesity, physical activity, and stress all play a part in psoriasis by upregulating chronic inflammatory responses.^1^, ^29^ Indigenous people in the Auaris region of Brazil were reported to have no cases of psoriasis, likely due to abundant natural sunlight exposure and absence of urban lifestyle factors.^12^ In the Inuit population of Nuuk, patients with psoriasis had higher associations with comorbidities such as metabolic syndrome and cardiovascular diseases.^29^

### Cultural practices and skin phenotype

Cultural values, traditional therapies, and skin phenotype play an important role in ensuring accurate diagnosis and treatment of psoriasis in Indigenous populations. Women of African ancestry tend to experience more severe forms of scalp psoriasis due to Afro-textured hair, hair care practices (chemical relaxers, braids, weaves), and longer washing intervals, all of which can complicate adherence to topical treatment regimens.^38^ In Tigray, Ethiopia, a case series reported frequent use of Indigenous therapies, including Hijama (wet cupping) and traditional plants such as Kigelia africana.^9^ These treatments have inconclusive dermatological outcomes, and in some cases were associated with disease flares or post-inflammatory hyperpigmentation. Access to conventional therapies is limited, with most patients restricted to topical corticosteroids, salicylic acid, or methotrexate.^15^ Traditional Asian practices such as cupping, coining, moxibustion, and herbal remedies may also exacerbate cutaneous disease activity.^38^

One of the most important factors that complicates accurate estimation of prevalence is diagnostic accuracy, particularly in skin of colour. In Indigenous populations with darker skin phototypes, psoriasis may present with atypical features, leading to underdiagnosis or misdiagnosis. Erythema in Fitzpatrick type VI skin may appear brown or violaceous, complicating assessment and contributing to underestimation of Psoriasis Area and Severity Index (PASI) scores.^32^, ^38^ Post-inflammatory hyper- and hypopigmentation are also more prominent in these populations, further complicating clinical evaluation. Among Inuit populations, psoriasis often presents as thin plaques resembling nummular eczema, which may delay diagnosis and result in more advanced disease at presentation.^10^, ^15^ Delayed recognition not only impacts treatment outcomes but also increases the risk of progression to severe disease, for which systemic therapies may be unavailable or difficult to access in remote communities.

### Reduced access to dermatological services and research

A major limitation of this review is that much of the available data consists of anecdotal descriptions or results from small field studies with limited population numbers. The scarcity of large-scale epidemiological data of indigenous populations is influenced by the underlying issues of reduced access to dermatological services, underrepresentation in clinical trials, and challenges in diagnostic accuracy.

In many low-resource settings, empowering remote and indigenous communities to access mainstream dermatological services can be challenging. In rural regions of Africa, the diagnosis and management of skin conditions, including psoriasis, are often provided by community health workers or non-medical individuals.^41^ This leads to misdiagnosis of psoriasis (most commonly as tinea corporis) and delays to appropriate treatment, hence resulting in disease progression and greater morbidity.^32^ Access barriers are also present in high-income nations. In Australia, only 4% of dermatologists are based in rural and remote areas and just 2% in regional areas, despite these areas being home to a large proportion of Indigenous communities.^42^ Historical and intergenerational trauma further compound these challenges. Among Aboriginal Australians, cultural attitudes toward skin disease, combined with rurality and the enduring legacy of colonisation, have been cited as barriers to engagement with mainstream dermatological services.^8^

Research gaps also contribute to underrepresentation. Clinical trials and observational studies frequently omit detailed reporting of the racial or ethnic background of participants, limiting the capacity to accurately assess disease burden in indigenous communities.

While biological, environmental, and cultural factors likely contribute to the lower prevalence of psoriasis in Indigenous communities, the lack of standardised, high-quality epidemiological data remains a significant barrier to understanding the true global burden of disease. Improving data collection and reporting practices is, therefore, critical to addressing inequities and informing public health policy.

## Conclusion

Based on the available data and proposed biological, environmental, cultural and social explanations, psoriasis appears to be less frequent in Indigenous populations; however, the current evidence is limited, scarce, and potentially unreliable. Significant challenges remain in ensuring timely diagnosis and equitable access to dermatological care, particularly for Indigenous peoples in remote settings.

To address these gaps, future research should prioritise large-scale, population-based epidemiological studies that employ standardised diagnostic criteria and explicitly include Indigenous participants. Improving the quality and inclusivity of data collection is essential to clarify the true global burden of psoriasis and to guide equitable healthcare planning and resource allocation.

## ORCID ID

Yebin Yang: 0009-0001-6623-135X

Sangho Lee: 0009-0004-4846-5381

Francis Yi Xing Lai: 0000-0003-3976-1432

Ricardo Romiti: 0000-0003-0165-3831

## Financial support

The authors received no specific funding for this study.

## Authors’ contributions

Yebin Yang: Data collection, analysis and interpretation; preparation and writing of the manuscript; statistical analysis; study conception and planning.

Sangho Lee: Approval of the final version of the manuscript; manuscript critical review; study conception and planning.

Francis Yi Xing Lai: Approval of the final version of the manuscript; effective participation in research orientation; manuscript critical review; study conception and planning.

Ricardo Romiti: Approval of the final version of the manuscript; manuscript critical review; study conception and planning.

## Research data availability

The entire dataset supporting the results of this study was published in this article.

## Conflicts of interest

None declared.
